# Reduced Anticipatory Pleasure in Early-Onset Severe Cannabis Use Disorder: A Retrospective, Cross-Sectional Study

**DOI:** 10.3390/ejihpe16100152

**Published:** 2026-09-27

**Authors:** Maria Pepe, Francesco Traccis, Giuseppe Mandracchia, Marco Massetti, Martina Platia, Isabella Panaccione, Raffaella Franza, Gabriele Sani, Marco Di Nicola

**Affiliations:** 1 Department of Neurosciences, Head-Neck and Chest, Section of Psychiatry, Fondazione Policlinico Universitario Agostino Gemelli IRCCS, Largo Agostino Gemelli 8, 00168 Rome, Italy; maria.pepe@unicatt.it (M.P.); gabriele.sani@unicatt.it (G.S.); 2 Department of Neuroscience, Section of Psychiatry, Università Cattolica del Sacro Cuore, Largo Francesco Vito 1, 00168 Rome, Italy; 3 Service of Clinical Psychology, Fondazione Policlinico Universitario Agostino Gemelli IRCCS, Largo Agostino Gemelli 8, 00168 Rome, Italy; 4 Department of Mental Health, ASL Roma 1, Via Plinio 31, 00193 Rome, Italy

**Keywords:** addiction, cannabinoids, motivational dysfunction, reward anticipation, youth

## Abstract

**Background:** Cannabis is the most widely used illicit substance, particularly among youth. Earlier onset is associated with poorer clinical trajectories in cannabis use disorder (CUD), but its relationship with hedonic–motivational components remains unclear. **Methods:** Outpatients with severe CUD (*n* = 121) were stratified by age at onset (<18 vs. ≥18 years) and compared on hedonic–motivational functioning using the Snaith–Hamilton Pleasure Scale (SHAPS) and the Temporal Experience of Pleasure Scale (TEPS). Healthy subjects (*n* = 112) served as a reference group. **Results:** CUD groups were comparable in clinical characteristics and cannabis use patterns. TEPS-Anticipatory scores were lower in patients with onset before age 18 (*p* < 0.001) and remained associated with onset group after adjustment for gender, education, and CUD duration (*p* < 0.001). When healthy subjects were included, significant group effects were observed (*p* < 0.001). Post hoc comparisons confirmed reduced TEPS-Anticipatory scores in early-onset vs. both later-onset and control groups, and higher SHAPS scores in both CUD groups vs. controls. **Conclusions:** CUD onset before age 18 is associated with reduced anticipatory pleasure, suggesting a greater involvement of anticipatory than other dimensions of hedonic functioning. Age at onset may therefore help identify patients with greater motivational vulnerability and inform early, targeted prevention and intervention strategies.

## 1. Introduction

Cannabis is the most widely used illicit drug worldwide (UNODC, 2025) and in Europe, particularly among adolescents and young adults (EUDA, 2026). Consistently, current cannabis use among 15–16-year-old students in Italy reaches 8.6%, exceeding the European average of 5% (ESPAD, 2024). Although cannabis use is often intermittent or recreational, a non-negligible proportion of users develop a cannabis use disorder (CUD), a condition associated with clinically significant impairment and increased psychopathological vulnerability (Budney, 2019; Connor et al., 2021).

CUD can lead to several adverse outcomes, including affective dysregulation, cognitive dysfunction, reduced psychosocial functioning, and an increased risk of psychiatric comorbidity, particularly within the psychotic spectrum (Hjorthøj et al., 2021; Murray et al., 2017; Sagar & Gruber, 2025). Age at onset represents a well-established marker of clinical vulnerability in substance use disorders (Butterworth et al., 2014), where an earlier initiation is typically associated with greater disorder severity, more persistent use patterns, and poorer long-term outcomes (Solmi et al., 2022; Wagner & Anthony, 2002).

In the context of cannabis use, early exposure during a critical neurodevelopmental period such as adolescence has been linked to more pronounced neurobiological and functional alterations (Macedo et al., 2024; Rubino & Parolaro, 2016; Volkow et al., 2019). The primary psychoactive component of cannabis, Δ9-tetrahydrocannabinol (THC), exerts its effects mainly through the endocannabinoid system, which plays a key modulatory role in synaptic plasticity and dopaminergic neurotransmission (Andre et al., 2016; Volkow et al., 2017). Through its partial agonism at cannabinoid CB1 receptors, THC can indirectly modulate mesolimbic dopaminergic signaling, a mechanism central to reward processing and reinforcement learning (Caballero & Tseng, 2012; Hirvonen et al., 2012). Experimental and human studies suggest that repeated exposure to cannabinoids, particularly when occurring early in development, may interfere with the maturation of mesolimbic reward pathways (Rubino & Parolaro, 2016), thereby inducing persistent neuroadaptations (Bloomfield et al., 2014, 2016; Volkow et al., 2017).

Alterations in reward processing and motivation have been documented in individuals with chronic cannabis use and have historically been conceptualized under the construct of an “amotivational syndrome”, characterized by apathy, reduced initiative, diminished goal-directed behavior, and decreased responsiveness to rewarding stimuli (Barnwell et al., 2006). More recent models have refined the conceptualization of hedonic experience, distinguishing between anticipatory aspects of reward, which support motivation toward future goals, and consummatory pleasure, which reflects the immediate hedonic experience during reward consumption (Gard et al., 2006; Moccia et al., 2018).

Consistent with this multidimensional conceptualization, research on cannabis use has examined different components of reward and hedonic functioning using self-report, behavioral, and neuroimaging measures, yielding heterogeneous findings across reward subprocesses and study populations (Beyer et al., 2024; Skumlien et al., 2021). Studies employing self-report measures of hedonic capacity, including instruments assessing anticipatory and consummatory pleasure, suggest that alterations in pleasure experience may be related to cannabis involvement, although associations appear to vary according to developmental and clinical characteristics, as well as to whether cannabis involvement is indexed in terms of use frequency or problematic use (Cassidy et al., 2012; Leventhal et al., 2017; Lopez-Vergara et al., 2019).

Importantly, although age at initiation of cannabis use has been explored in relation to reward-related outcomes, evidence regarding this association remains limited and inconsistent across different reward sub-processes (Beyer et al., 2024). Moreover, considerably less is known about whether the age at onset of CUD itself is associated with alterations in distinct hedonic–motivational components. Indeed, it is unclear whether CUD with onset before age 18 is selectively associated with deficits in specific motivational processes beyond the general clinical severity observed in early-onset trajectories (Budney, 2019; Connor et al., 2021). Against this background, the present study aimed to investigate the association between age at onset of CUD and hedonic–motivational functioning in a clinical sample of patients with severe CUD, with the hypothesis that an earlier age at onset would be associated with altered hedonic–motivational functioning, while exploring whether such alterations differentially involved anticipatory and consummatory components of pleasure.

## 2. Materials and Methods

### 2.1. Participants

Outpatients consecutively referred to the “Centro Psichiatrico Integrato per la ricerca, la prevenzione e la cura delle Dipendenze” (CePID) at the Department of Psychiatry of Fondazione Policlinico Universitario “Agostino Gemelli” IRCCS in Rome, between January 2021 and March 2024, for substance-related problems were retrospectively screened for inclusion. Subjects were considered eligible if they were of either gender and had a diagnosis of severe Cannabis Use Disorder (CUD) according to the DSM-5 criteria (APA, 2013). The primary diagnosis and the presence of any psychiatric comorbidities were evaluated through the Italian version of the Structured Clinical Interview for DSM-5 Disorders, Clinician Version, and Personality Disorders. Further inclusion criteria included fluency in spoken and written Italian. Participants were excluded if they presented acute intoxication at the time of assessment, major medical disorders, or any conditions that could impair psychometric assessment, such as active psychotic features, organic brain syndromes, neurocognitive disorders, or evidence of global cognitive impairment, operationalized as a Mini-Mental State Examination score of <26.

The sample was stratified a priori by age at onset of CUD (before age 18 vs. 18 years or older) to distinguish onset occurring within adolescence from onset occurring thereafter. Age at CUD onset was defined as the retrospectively ascertained age at which patients first met DSM-5 diagnostic criteria for CUD, as documented during the clinical assessment, integrating patient report with collateral information from family members or caregivers, when available and with the patient’s consent. Although neurodevelopmental processes extend into young adulthood, this categorization is consistent with models identifying adolescence as a sensitive period for substance-related vulnerability, a notion supported by evidence indicating this age range as critical for prefrontal cortex maturation and dopaminergic circuitry development (Paus et al., 2008; Volkow et al., 2019). Further, the selected cut-off is consistent with widely adopted operational definitions of early-onset CUD (Connor et al., 2021), supporting its relevance for examining the association between early cannabinoid exposure and hedonic–motivational functioning.

Healthy subjects (HS) were additionally recruited from the community as a reference group for the comparison of hedonic experience, without any financial or other participation incentive. They were volunteers screened for current or previous psychiatric disorders, including substance abuse or dependence, and related current or previous treatment (psychotherapy and/or psychopharmacological treatment); a positive response constituted an exclusion criterion.

The sample size was determined by the number of eligible individuals available during the study period, and no a priori sample-size calculation was performed. The study protocol received local ethical approval, and written informed consent was obtained from all participants prior to inclusion.

### 2.2. Data Collection and Psychometric Assessment

Clinical records were retrospectively reviewed for extraction of sociodemographic, clinical, substance-use, and psychometric data, which had been collected as part of routine clinical practice through structured clinical interviews, self-report measures, and toxicological assessments, as detailed below. Psychometric assessment was performed after at least four weeks of sustained abstinence from cannabis and, when applicable, other substances of abuse, following an integrated pharmacological and psychotherapeutic detoxification program. Cannabis use and abstinence were established using information from clinician interviews, self-reports (including a TimeLine FollowBack calendar), and qualitative urine toxicology screenings. Detailed information on cannabis use comprised duration of use, frequency of consumption, average daily quantity, and type of cannabinoids used. Cannabis product type was based on participants’ self-report during the clinical interview; THC and CBD concentrations were not analytically verified.

Hedonic-motivational functioning was assessed using the validated Italian versions of the Temporal Experience of Pleasure Scale (TEPS; Gard et al., 2006) and the Snaith–Hamilton Pleasure Scale (SHAPS). The TEPS is an 18-item self-report scale rated on a 6-point Likert scale (1–6), designed to assess trait hedonic capacity and individual differences in the experience of pleasure, with higher scores indicating greater hedonic capacity. The questionnaire shows a multifactorial structure comprising three dimensions of pleasure: ‘anticipatory’, a motivational, future-oriented component related to the expectation of rewarding experiences (“wanting”); ‘contextual anticipatory’, anchored to specific situations or contextual conditions perceived as pleasurable; ‘consummatory’, the in-the-moment experience of pleasure during rewarding activities (“liking”). Factor scores were computed according to the three-factor structure and item allocation reported in the Italian validation study (Stratta et al., 2011), comprising anticipatory, contextual anticipatory, and consummatory pleasure dimensions. The Italian version has demonstrated satisfactory psychometric properties. In the present sample, internal consistency was good for the overall TEPS (Cronbach’s α = 0.806; McDonald’s ω = 0.808), while coefficients for the anticipatory, contextual anticipatory, and consummatory dimensions were α/ω = 0.752/0.757, 0.563/0.564, and 0.634/0.644, respectively.

Anhedonia was additionally assessed using the SHAPS, a self-report instrument comprising 14 items covering common pleasurable experiences across four domains (interests and pastimes, social interaction, sensory experiences, and food/drink). Respondents rate their level of agreement based on recent experience, and responses are summed to yield a total score ranging from 0 to 14, with higher scores indicating greater anhedonia and a commonly used cut-off of ≥3 for clinically relevant symptomatology (Snaith et al., 1995). The SHAPS has demonstrated good reliability and validity in both clinical and non-clinical populations, including the Italian validation (Martino et al., 2018). In the present sample, internal consistency was α = 0.658 and ω = 0.662. It is primarily designed to assess hedonic tone related to experienced pleasure and does not directly measure anticipatory or motivational components of reward processing (Mittmann et al., 2025; Trøstheim et al., 2020). Here, continuous SHAPS scores were used in all analyses.

### 2.3. Statistical Analysis

Descriptive data were summarized as the number of participants and percentage (%) or mean ± standard deviation (M ± SD) for categorical and continuous variables, respectively. Univariate comparisons between groups on sociodemographic, clinical, and psychometric variables were conducted using the independent-samples t-test for continuous variables or the Chi-squared test (or Fisher’s exact test, when appropriate) for dichotomous variables.

Linear regression analyses were performed to examine the association between age at CUD onset and hedonic–motivational functioning within the clinical sample. All three TEPS dimensions were examined and are reported both in the univariate comparisons within the clinical sample and in the analyses including healthy subjects described below; no outcome was excluded from the analytical plan based on observed *p*-values. The adjusted regression was applied to TEPS-Anticipatory, the only dimension associated with onset group in those analyses. Age at onset group (before-18 vs. 18-or-older onset CUD) was entered as the main factor, adjusting for gender, educational level, and CUD duration; these covariates were selected a priori on substantive clinical and sociodemographic grounds (Heinze et al., 2018), with CUD duration accounting for cumulative cannabis exposure and reducing the risk that the investigated association reflected illness duration rather than onset timing. Current age was not included in the same model because age at onset, CUD duration, and current age are conceptually and mathematically interrelated, raising concerns regarding redundancy and collinearity. Model diagnostics included assessment of residual normality, homoscedasticity, and multivariate outliers. Alongside the a priori categorical analysis, age at CUD onset was additionally modeled as a continuous predictor within the same covariate-adjusted model, and a quadratic term for mean-centered age at onset was tested to examine departures from linearity. Collinearity diagnostics were examined using variance inflation factors (VIF), with values of ≤3 considered acceptable.

Healthy participants were included as a reference group to test between-group differences in specific hedonic-motivational dimensions and global anhedonia. Multivariate analysis of covariance (MANCOVA) was conducted with the three TEPS subscales as dependent variables and group (before-18-onset CUD, 18-or-older-onset CUD, healthy subjects) as the between-subjects factor. Age and gender were included as covariates in all multivariable models due to baseline differences between groups. Homogeneity of covariance matrices and multivariate normality were assessed prior to the MANCOVA, and Pillai’s Trace was additionally inspected because of its relative robustness to departures from multivariate normality. When a significant multivariate effect was detected, we performed a series of one-way analyses of covariance (ANCOVAs), with the same covariates, to test differences between groups on dependent variables. Similarly, an ANCOVA model was performed to investigate between-group differences in SHAPS scores. For outcomes showing significant group effects, Bonferroni-adjusted pairwise comparisons were performed. Lastly, exploratory Pearson’s correlation analyses were conducted to examine the association between global anhedonia (SHAPS total score) and hedonic–motivational dimensions (TEPS subscales).

To account for multiple testing across the three TEPS dimensions, a Bonferroni-adjusted significance threshold of α = 0.017 was applied (Bender & Lange, 2001).

No imputation was performed at either the item or the scale level, and no prorating was applied to incomplete questionnaires. All variables entering the linear regression, MANCOVA, and ANCOVA models were complete, including all TEPS and SHAPS scores; these analyses were therefore conducted on the full samples (*n* = 121 for the within-CUD models; *n* = 233 for the models including healthy subjects), with no observations excluded. Statistical significance was set at *p* < 0.05 unless otherwise specified. All analyses were performed using IBM SPSS Statistics for Windows, v. 29.

## 3. Results

The clinical records of 285 patients referred during the study period were retrospectively screened for eligibility. After excluding those who did not provide informed consent (*n* = 75) and participants who did not satisfy inclusion criteria (*n* = 89), 121 Caucasian subjects with severe CUD were included (File S1). Sociodemographic, clinical, and psychometric characteristics of the sample, together with available characteristics of the healthy reference group, are summarized in Table 1.

Mean age at cannabis use disorder onset was 15.2 ± 1.38 years for the before-18-onset CUD group, and 21.1 ± 2.11 years for the 18-or-older-onset CUD group. Regarding cannabis use patterns, patients reported frequent daily consumption (mean 3.72 ± 2.82 joints per day), without differences between groups (*p* = 0.065). Hashish/cannabis resin was the most commonly reported cannabis product, followed by marijuana and lower-THC products. At the time of assessment, 33.9% of patients were receiving psychopharmacological treatment, without significant differences between before-18-onset (*n* = 27) and 18-or-older-onset (*n* = 14) CUD groups (*p* = 0.449). A family history of substance use disorders was reported by 24.0% of patients, with comparable rates between before-18 and 18-or-older-onset groups (25.9% vs. 19.4%, respectively; *p* = 0.448). Psychiatric comorbidities were common across the sample (47.1%) and similarly distributed between groups (*p* = 0.678). Overall, the most frequent conditions were additional alcohol/substance use disorders (28.1%), neurodevelopmental (21.5%) and mood disorders (20.7%; 18.8% vs. 25% in the before-18-onset and 18-or-older-onset CUD groups, respectively; Fisher’s exact *p* = 0.467), including depressive disorders (15.7% overall; 14.1% vs. 19.4%; Fisher’s exact *p* = 0.585) and bipolar spectrum disorders (5% overall; 4.7% vs. 5.6%; Fisher’s exact *p* = 1.000). Other comorbidities included anxiety disorders (14.9%), personality disorders (10.7%), and gambling disorder (4.9%). Schizophrenia spectrum disorders (2.5%), obsessive–compulsive disorder (1.6%), and feeding/eating disorders (1.6%) were comparatively uncommon.

Among psychometric measures, TEPS-Anticipatory scores significantly differed between before-18 and 18-or-older-onset CUD at univariate analysis (*p* < 0.001), and this association remained significant in the linear regression model adjusted for gender, educational level, and CUD duration (*p* < 0.001, R^2^ = 0.129; Adjusted R^2^ = 0.091, AIC = 818). Estimated marginal means (SE) indicated lower TEPS-Anticipatory scores in patients with before-18-onset CUD [30.7 (0.76); 95% CI: 29.2–32.2] than in those with 18-or-older-onset CUD [35.9 (1.20); 95% CI: 33.6–38.3]. Model diagnostics did not indicate relevant violations of residual normality (Shapiro–Wilk *p* = 0.070) or homoscedasticity (Breusch–Pagan *p* = 0.088), with low collinearity (VIFs = 1.03–1.13) and no multivariate outliers (no observations exceeded the prespecified *p* < 0.001 Mahalanobis-distance threshold). Regression coefficients and model parameters are reported in Table 2.

In a sensitivity analysis modeling age at CUD onset as a continuous predictor within the same covariate-adjusted model, the association with TEPS-Anticipatory scores was in the same direction as the categorical contrast (B = 0.393, *p* = 0.034). Adding a quadratic term for mean-centered age at onset provided no evidence of departure from linearity (B = −0.011; *p* = 0.635; ΔR^2^ = 0.0018).

To contextualize these findings, a group of 112 healthy subjects was included as a reference population. As shown in Table 1, healthy subjects differed from the overall clinical sample in age and gender distribution (both *p* < 0.001). Accordingly, age and gender were included as covariates in subsequent MANCOVA and ANCOVA models.

After controlling for age and gender, the MANCOVA reported a significant global effect of group (before-18-onset CUD vs. 18-or-older-onset CUD vs. healthy subjects) on TEPS subscales (Wilks’ Lambda = 0.77, F(6,452) = 10.61, *p* < 0.001), whereas no significant multivariate effects were observed for age (Wilks’ Lambda = 0.99, F(3,226) = 0.21, *p* = 0.895) or gender (Wilks’ Lambda = 0.98, F(3,226) = 1.39, *p* = 0.246). Box’s M did not indicate heterogeneity of covariance matrices (*p* = 0.110); multivariate normality was not fully supported (*p* = 0.002), but the group effect was confirmed by Pillai’s trace (V = 0.232, F(6,454) = 9.95, *p* < 0.001).

To control for multiple comparisons across the three TEPS subscales, a Bonferroni-adjusted significance threshold was applied (α = 0.017), under which only TEPS-Anticipatory scores remained significant. Accordingly, a separate ANCOVA including the same covariates was conducted and is fully reported in Table 3, together with Bonferroni-adjusted post hoc comparisons, while estimated marginal means and their CIs are illustrated in Figure 1.

Overall, TEPS-Anticipatory scores were sufficiently comparable across groups, as supported by an additional multigroup confirmatory factor analysis showing measurement invariance for this dimension (CFI = 0.986, RMSEA = 0.029, SRMR = 0.062), with no deterioration in model fit after progressively constraining factor loadings (metric invariance: CFI = 1.000, RMSEA = 0.000, SRMR = 0.072) and item intercepts (scalar invariance: CFI = 1.000, RMSEA = 0.000, SRMR = 0.080).

No significant group effects were observed for TEPS-Contextual Anticipatory (F = 0.42, unadjusted *p* = 0.659, η^2^p = 0.004) or TEPS-Consummatory scores (F = 1.64, unadjusted *p* = 0.196, η^2^p = 0.014; see File S2 for full results).

Although SHAPS did not differentiate before-18 from 18-or-older-onset CUD within the clinical sample, to further characterize overall anhedonia, an ANCOVA including healthy subjects was performed, with age and gender as covariates, which is reported in Table 4 together with Bonferroni-adjusted pairwise comparisons. Estimated marginal means indicated higher levels of anhedonia in both before-18-onset [5.51 (SE = 0.22); 95% CI: 5.07–5.95] and 18-or-older-onset CUD [5.15 (SE = 0.33); 95% CI: 4.49–5.80] compared with healthy subjects [2.41 (SE = 0.20); 95% CI: 2.02–2.80].

Given that education was included as a covariate in the within-clinical-sample regression model, sensitivity analyses were performed by additionally adjusting for this variable in the MANCOVA and ANCOVA models including healthy subjects. This additional adjustment did not materially alter the pattern of findings (see File S3).

Exploratory correlation analyses showed significant negative associations between SHAPS and TEPS-Anticipatory (r = −0.322, *p* < 0.001), and TEPS-Total score (r = −0.212, *p* = 0.001; see File S4 for complete correlation results).

## 4. Discussion

In this sample of patients with severe cannabis use disorder (CUD), an earlier age at onset (before age 18) was associated with lower self-reported anticipatory pleasure, whereas other facets of hedonic experience did not show the same pattern. Notably, patients with onset before age 18 and those with onset at 18 years or older were largely comparable across clinical characteristics and cannabis use. Importantly, the association between earlier onset and lower TEPS-Anticipatory scores remained significant after adjustment for gender, educational level, and CUD duration. Taken together, these findings suggest that onset before age 18 may identify a subgroup of patients with severe CUD characterized by lower anticipatory pleasure, potentially beyond differences in other clinical characteristics.

The clinical profile of the sample, predominantly young adult males with frequent daily cannabis use and a preference for resin-based products, mirrors current epidemiological patterns of consumption (Connor et al., 2021; EUDA, 2026; UNODC, 2025). Cannabis use remains prevalent among male adolescents and young adults, although gender differences have progressively narrowed (Di Nicola et al., 2017; ESPAD, 2024; EUDA, 2026). In parallel, the increasing availability of high-potency cannabis products, especially resin with elevated THC concentrations (EUDA, 2026; Freeman et al., 2021), has been linked to adverse neuropsychiatric outcomes, particularly when exposure occurs during developmentally sensitive periods (Hambly Lapointe et al., 2026; Hurd, 2025; Wijayendran et al., 2018).

The finding of reduced anticipatory pleasure in individuals with earlier CUD onset is consistent with contemporary models of reward processing, which distinguish anticipatory, motivational components of pleasure from consummatory aspects (Gard et al., 2006). Anticipatory reward processing, often conceptualized as “wanting”, supports goal-directed behavior and effort allocation, whereas consummatory pleasure (“liking”) refers to the hedonic experience during reward consumption (Volkow et al., 2017). Accordingly, the different pattern observed in the TEPS subscale scores points to a potentially greater involvement of anticipatory aspects of pleasure than of overall hedonic functioning in relation to earlier CUD onset. This interpretation is consistent with a recent systematic review framing CUD as a condition characterized by motivational dysregulation, with altered goal pursuit and reduced engagement with non-drug rewards as clinically relevant features (Mishra et al., 2026). Against the heterogeneous findings previously reported across different measures of reward and hedonic functioning in cannabis users (Skumlien et al., 2021), our findings further suggest that age at CUD onset may contribute to characterizing variability in hedonic–motivational functioning among individuals with severe CUD.

The observed reduction in anticipatory pleasure may be interpreted in light of evidence implicating dopaminergic dysfunction in cannabis-related motivational alterations. Studies in chronic cannabis users have reported reduced striatal dopaminergic reactivity, altered reward-related neural activation, and blunted dopamine synthesis capacity, particularly during tasks involving reward anticipation rather than reward receipt (Rubino & Parolaro, 2016). These alterations have been associated with apathy and reduced motivational drive, even in the absence of marked changes in baseline dopamine receptor availability (Bloomfield et al., 2014), suggesting that cannabis-related deficits may primarily affect dynamic aspects of reward signaling rather than tonic hedonic function.

From a neurodevelopmental perspective, adolescence represents a critical period for the maturation of frontolimbic and mesocorticolimbic circuits involved in reward processing and behavioral regulation. During this phase, dopaminergic systems show increased plasticity and responsivity to reinforcing stimuli, while prefrontal regulatory systems are still developing (Paus et al., 2008). Exposure to cannabinoids during this period may interfere with the maturation of these circuits through modulation of GABAergic and glutamatergic transmission and indirect effects on dopaminergic signaling (Freeman et al., 2021). Importantly, the magnitude of these effects may depend on THC exposure, as emerging translational evidence indicates dose-related consequences during adolescence (Hurd, 2025; Tseng & Molla, 2025). In nonhuman primates, prolonged exposure during this sensitive period produced persistent alterations in ventral striatal connectivity and dose-dependent disruption of reward sensitivity and motivation, although direct evidence that higher THC potency specifically produces greater blunting of ventral striatal activation during reward anticipation remains limited (Kangas et al., 2025). Persistent neuroadaptations, including CB1 receptor downregulation and altered dopaminergic responsivity, may contribute to long-term changes in motivational salience attribution (Caballero & Tseng, 2012; Hirvonen et al., 2012; Rubino & Parolaro, 2016).

Within this framework, alterations in dopaminergic signaling may be relevant for anticipatory components of reward processing, which are closely linked to motivational salience attribution and future-oriented behavior (Volkow et al., 2017). Altered dopaminergic function has been associated with reduced reward anticipation (Dubol et al., 2018), while neuroimaging studies have shown that blunted ventral striatal responses to anticipated rewards during adolescence are linked to increased vulnerability to later substance use (Büchel et al., 2017). Moreover, developmental refinement of reward anticipation and reward receipt processes during adolescence (Cao et al., 2019), together with individual variability in dopaminergic function associated with susceptibility to substance misuse (Baker et al., 2019), further supports the relevance of this developmental period. Prospective evidence suggests that reward-related characteristics may precede substance involvement. In substance-naïve children, nucleus accumbens activation during reward anticipation predicted subsequent substance-use initiation (Cope et al., 2019), indicating that individual differences in reward processing can be present before substance exposure. Conversely, longitudinal evidence also supports a potential contribution of cannabis exposure to subsequent changes in reward processing. Greater marijuana use has been associated with subsequent reductions in nucleus accumbens activation during reward anticipation (Martz et al., 2016), suggesting that alterations in anticipatory reward signaling may also emerge or become more pronounced over the course of cannabis use. More recent evidence further suggests that reward-related profiles associated with substance use may vary across developmental stages and patterns of use, leaving the temporal relationship between reward function and substance involvement unresolved (Martz et al., 2021). Taken together, pre-existing reward-related characteristics and cannabis-associated neuroadaptations may represent complementary rather than mutually exclusive processes, although the cross-sectional design of the present study does not allow their relative contribution to the observed reduction in anticipatory pleasure to be determined.

Similar alterations in reward anticipation have been described across several psychiatric conditions characterized by motivational dysfunction, including major depression, schizophrenia spectrum disorders, and other substance use disorders (Bloomfield et al., 2014; Hinckley et al., 2024). In these conditions, impairments in reward anticipation have been associated with apathy, reduced initiative, and diminished goal-directed behavior, even when consummatory pleasure remains relatively preserved (Hinckley et al., 2024). Furthermore, anhedonia has been shown to be closely linked to substance use disorders across different levels of severity and in the presence of psychiatric comorbidities (Stull et al., 2022), and to dynamically interact with mood states in influencing cannabis use patterns (Collins et al., 2024). Importantly, in this sample, psychiatric comorbidities were present but similarly distributed between groups, suggesting that the observed differences in anticipatory pleasure are unlikely to be driven by differential comorbidity burden. From a broader transdiagnostic perspective, alterations in reward anticipation have been proposed as a shared dimension of vulnerability contributing to functional impairment across diagnostic categories (Hinckley et al., 2024).

In addition to differences in anticipatory pleasure, these findings provide insight into broader hedonic functioning as assessed by the SHAPS. Although descriptively higher SHAPS scores were observed in patients with onset before age 18, this difference did not reach statistical significance within the clinical sample. In contrast, both CUD groups showed significantly higher levels of anhedonia than healthy subjects, suggesting that reduced global hedonic tone may characterize severe CUD more broadly, without the same differentiation according to age at onset observed for anticipatory pleasure. These findings suggest that global anhedonia and anticipatory pleasure may capture partially distinct aspects of reward functioning, consistent with previous work emphasizing the multidimensional nature of reward-related alterations in cannabis use (Skumlien et al., 2021). While SHAPS scores indicate a broader reduction in hedonic tone across individuals with severe CUD, lower TEPS-Anticipatory scores appear associated with earlier disorder onset. Consistently, exploratory analyses revealed a moderate negative correlation between SHAPS and TEPS-Anticipatory scores, suggesting that these constructs are related but not interchangeable, in line with multidimensional models of anhedonia and reward processing (Gard et al., 2006; Mittmann et al., 2025; Trøstheim et al., 2020).

Clinically, these findings may have implications for the characterization of hedonic–motivational functioning in severe CUD and for the identification of potentially relevant treatment targets. In particular, reduced anticipatory pleasure may be associated with goal-directed behavior and motivational engagement, dimensions relevant to treatment adherence and recovery processes (Budney, 2019; Gates, 2016). Interventions that enhance engagement with alternative, non-drug rewards and support goal-directed behavior, including contingency management and behavioral activation approaches, may warrant consideration, particularly when reduced anticipatory pleasure is evident (Gates, 2016; Haney et al., 2023; Mishra et al., 2026). More broadly, the findings support prevention strategies aimed at delaying the onset of problematic cannabis use during adolescence (Wagner & Anthony, 2002).

Several limitations should be acknowledged when interpreting these findings. The cross-sectional design precludes determination of whether reduced self-reported anticipatory pleasure reflects a pre-existing vulnerability, neuroadaptations associated with cannabis exposure, or a combination of these processes. The use of self-report measures may not fully capture behavioral or neurobiological indices of reward processing (Hinckley et al., 2024). The inclusion of treatment-seeking individuals with severe CUD recruited in a specialized clinical setting may limit generalizability to community samples or less severe forms of cannabis-related problems. In addition, detailed information on cannabis composition, including THC potency, was not systematically available. Age at onset and cannabis-use patterns were retrospectively ascertained and may be subject to recall and measurement bias. Although groups did not significantly differ in the prevalence of mood and depressive disorders, the lack of dedicated measures of current affective symptoms related to hedonic experience precludes fully excluding their potential confounding effect on reward processing. Dedicated dimensional measures of cannabis withdrawal severity were likewise unavailable; although psychometric assessment followed at least four weeks of sustained abstinence, longer-lasting substance-related effects on hedonic measures cannot be excluded. Moreover, although the analyses were adjusted for relevant covariates, the contribution of unmeasured common-liability factors such as early-life adversity, familial and socioeconomic aspects, or neurodevelopmental vulnerabilities cannot be excluded (Verweij et al., 2013). Finally, as HS were recruited from the community as a non-matched healthy reference sample, the potential influence of differences in recruitment source, as well as in residual between-group differences on comparisons of hedonic measures, cannot be fully excluded.

Despite these limitations, this study provides novel evidence on the association between age at onset of CUD and distinct components of hedonic–motivational functioning in a well-characterized clinical sample. The inclusion of both patients with onset before age 18 and those with onset at 18 years or older, together with a healthy reference population, allowed for a more nuanced interpretation of motivational alterations. The prespecified dichotomous categorization at age 18 should not be interpreted as evidence of a discrete neurodevelopmental threshold; however, modeling age at CUD onset continuously yielded similar results. Thus, the present data are consistent with a graded association between earlier CUD onset and lower self-reported anticipatory pleasure.

## 5. Conclusions

In this sample of patients with severe CUD, those with onset before age 18 reported lower anticipatory pleasure than those with onset at 18 years or older, and this difference persisted after adjustment for gender, education, and CUD duration. The association was more evident for anticipatory pleasure than for the other dimensions of hedonic experience. These findings suggest that age at CUD onset may contribute to variability in hedonic–motivational functioning. The direction of this association remains to be established, as reduced anticipatory pleasure may reflect pre-existing reward-related characteristics, cannabis-associated neuroadaptations, or a combination of these processes. Longitudinal studies incorporating behavioral and/or neurobiological measures of reward anticipation are needed to disentangle these possibilities and determine whether reward-related alterations may help characterize clinically relevant heterogeneity within CUD and inform prevention and intervention strategies targeting motivational processes.

## Figures and Tables

**Figure 1:**
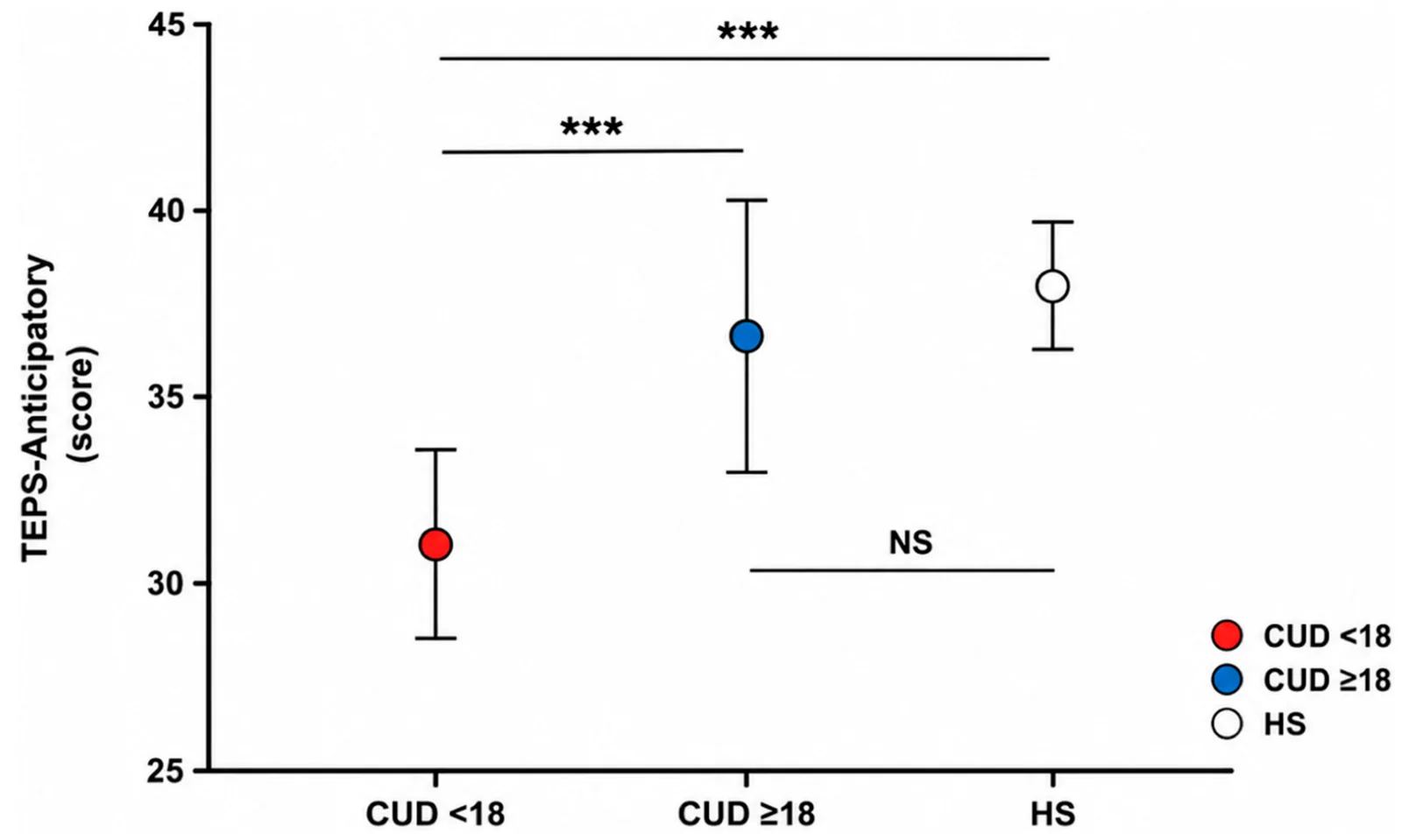
Between-group differences in anticipatory pleasure (TEPS-Anticipatory) with age and gender as covariates (ANCOVA). Adjusted estimated marginal means of TEPS-Anticipatory scores across groups. Error bars represent 95% confidence intervals. Abbreviations: ANCOVA, Analysis of Covariance; CUD, Cannabis Use Disorder; HS, Healthy Subjects; NS, not significant; TEPS, Temporal Experience of Pleasure Scale. Bonferroni-adjusted pairwise comparisons are indicated: *** *p* < 0.001.

**Table 1:** Sociodemographic, clinical, and psychometric characteristics of the sample.

*Characteristics (n, %; M ± SD)*	Total	Before-18-Onset CUD	18-or-Older-Onset CUD			HS	
**Overall**	121	85 (70.2)	36 (29.8)			112	
*Sociodemographic features*				**t/χ^2^/F**	***p***		***p***
**Age** (years)	28.2 ± 8.43	27.2 ± 8.15	30.4 ± 8.76	−1.94	0.054	24.9 ± 6.1	***
**Gender**				2.83	0.092		***
Male	104 (86)	76 (89.4)	28 (77.8)			46 (41.1)	
Female	17 (14)	9 (10.6)	8 (22.2)			66 (58.9)	
**Educational level** (years)	12.8 ± 2.56	12.3 ± 2.55	13.8 ± 2.33	−2.91	**0.004**	14.8 ± 2.8	***
**Occupation** (employed)	41 (33.9)	29 (34.1)	12 (33.3)	0.01	0.934	106 (94.6)	***
**Partner** (yes)	8 (6.6)	7 (8.2)	1 (2.8)	1.22	0.269	7 (6.3)	0.532
**Legal problems** (yes)	31 (25.6)	25 (29.4)	6 (16.7)	2.16	0.142	4 (3.6)	***
*Clinical data*							
**Duration of CUD** (years)	11.3 ± 8.04	12.1 ± 8.14	9.3 ± 7.59	1.74	0.084	-	
**Cannabis use**						-	
Days/week	6.4 ± 1.15	6.47 ± 1.09	6.26 ± 1.27	0.91	0.368		
Daily amount (grams)	1.11 ± 0.73	1.2 ± 0.76	0.93 ± 0.63	1.91	0.078		
**Age at first psychiatric access** (years)	26 ± 7.69	24.9 ± 6.76	28.2 ± 9.18	−1.96	0.052	-	
**Psychiatric hospitalizations** (yes)	22 (18.2)	17 (20)	5 (13.9)	0.63	0.426	-	
**Suicide attempts** (yes)	3 (2.48)	1 (1.18)	2 (5.6)	2.01	0.157	-	
**Psychiatric comorbidity** (yes)	57 (47.1)	39 (45.9)	18 (50)	0.16	0.678	-	
**Family history of psychiatric disorders** (yes)	57 (47.1)	36 (42.4)	21 (58.3)	2.59	0.107	10 (8.9)	***
**Polysubstance use** (yes)	34 (28.1)	25 (29.4)	9 (25)	0.24	0.622	-	
**BMI**	23.6 ± 3.82	23.2 ± 3.89	24.2 ± 3.65	−1.13	0.261	21.8 ± 2.6	***
**Tobacco smoking** (yes)	96 (79.3)	65 (76.5)	31 (86.1)	1.43	0.231	60 (53.6)	***
*Psychometric assessment*							
**SHAPS**	5.52 ± 1.36	5.65 ± 1.49	5.22 ± 0.93	1.58	0.117	2.29 ± 2.44	***
**TEPS**							
Anticipatory	32.3 ± 7.28	30.8 ± 7.2	35.8 ± 6.4	−3.60	**<0.001 ^#^**	38.3 ± 6.1	***
Contextual Anticipatory	19.8 ± 4.34	19.6 ± 4.2	20.5 ± 4.7	−1.05	0.295	20.2 ± 3.97	0.478
Consummatory	18.5 ± 3.60	18.1 ± 3.5	19.3 ± 3.9	−1.59	0.115	19 ± 3.53	0.221
Total score	70.5 ± 12	68.5 ± 11.3	75.5 ± 12.0	−3.06	**0.003**	77.5 ± 10.6	***

Significant results are in **bold**. Reported *p*-values are unadjusted, with ^#^ indicating results remaining significant after Bonferroni-adjusted significance threshold (α = 0.017, for the three TEPS dimensions). The *p*-values in the last column refer to comparisons between the overall clinical sample and healthy subjects, with *** *p* < 0.001 (continuous and categorical variables were compared using, respectively, Student’s *t*-test/Welch’s *t*-test, and χ^2^ test/Fisher’s exact test, as appropriate). Abbreviations: BMI, body mass index; CUD, cannabis use disorder; F, Fisher’s exact test; HS, healthy subjects; *M*, mean; SD, standard deviation; t, Student’s t; SHAPS, Snaith–Hamilton Pleasure Scale; TEPS, Temporal Experience of Pleasure Scale; χ^2^, chi-squared test.

**Table 2:** Linear regression analysis of TEPS-Anticipatory scores in patients with CUD.

*Predictor*	B	SE	95%CIs	*p*
**CUD onset group** (18-or-older vs. before-18)	5.24	1.46	2.35, 8.13	**<0.001**
*Covariate*				
CUD duration (years)	−0.03	0.08	−0.19, 0.13	0.744
Education	−0.41	0.26	−0.91, 0.10	0.116
Gender	2.09	1.84	−1.54, 5.73	0.256

Significant results are in **bold**. Abbreviations: B, unstandardized coefficients; CI, Confidence Intervals; CUD, Cannabis Use Disorder; *p*, statistical significance; SE, Standard Error; TEPS, Temporal Experience of Pleasure Scale. N = 121.

**Table 3:** Between-group differences in anticipatory pleasure (TEPS-Anticipatory) with age and gender as covariates (ANCOVA).

*Effect*	df	F	*p* *	η^2^p
**Group**	2228	20.72	**<0.001**	0.154
*Covariate*				
Age	1228	0.13	0.718	0.001
Gender	1228	2.46	0.118	0.011
*Pairwise comparisons*		**TEPS-Anticipatory**	***p_Bonferroni_***	
Group		MD (SE)		
**Before-18** vs. **18-or-older-onset CUD**		−4.8 (1.32)	**<0.001**	
**Before-18-onset CUD** vs. **HS**		−6.7 (1.06)	**<0.001**	
**18-or-older-onset CUD** vs. **HS**		−1.8 (1.33)	0.511	

* Unadjusted *p*-values (in **bold** if significant). Abbreviations: ANCOVA, Analysis of Covariance; CUD, Cannabis Use Disorder; df, degrees of freedom; HS, Healthy Subjects; MD, Mean Difference; *p*, statistical significance; SE, Standard Error; TEPS, Temporal Experience of Pleasure Scale. N = 233.

**Table 4:** Between-group differences in overall anhedonia (SHAPS) with age and gender as covariates (ANCOVA).

*Effect*	df	F	*p* *	η^2^p
**Group**	2228	53.00	**<0.001**	0.317
*Covariate*				
Age	1228	<0.001	0.994	<0.001
Gender	1228	3.12	0.079	0.013
*Pairwise comparisons*		**SHAPS**	***p_Bonferroni_***	
Group		MD (SE)		
**Before-18** vs. **18-or-older-onset CUD**		0.36 (0.39)	1.000	
**Before-18-onset CUD** vs. **HS**		3.10 (0.32)	**<0.001**	
**18-or-older-onset CUD** vs. **HS**		2.74 (0.40)	**<0.001**	

* Unadjusted *p*-values (in **bold** if significant). Abbreviations: ANCOVA, Analysis of Covariance; df, degrees of freedom; HS, Healthy Subjects; MD, Mean Difference; *p*, statistical significance; SE, Standard Error; SHAPS, Snaith–Hamilton Pleasure Scale. N = 233.

## Data Availability

The data presented in this study are available on request from the corresponding author due to institutional data-sharing policies.

## Supplementary Materials

The following supporting information can be downloaded at https://www.mdpi.com/article/10.3390/ejihpe16100152/s1. File S1: Participant flow. File S2: Full ANCOVA results for TEPS-Contextual Anticipatory and TEPS-Consummatory scores. File S3: Sensitivity analyses of between-group differences in hedonic measures, additionally adjusted for educational level. File S4: Complete SHAPS–TEPS correlation results.

## References

[B1-ejihpe-16-00152] American Psychiatric Association (APA) (2013). Diagnostic and statistical manual of mental disorders.

[B2-ejihpe-16-00152] Andre C. M., Hausman J. F., Guerriero G. (2016). Cannabis sativa: The plant of the thousand and one molecules. Frontiers in Plant Science.

[B3-ejihpe-16-00152] Baker T. E., Castellanos-Ryan N., Schumann G., Cattrell A., Flor H., Nees F., Banaschewski T., Bokde A., Whelan R., Buechel C., Bromberg U., Orfanos D. P., Gallinat J., Garavan H., Heinz A., Walter H., Brühl R., Gowland P., Paus T., the IMAGEN consortium (2019). Modulation of orbitofrontal-striatal reward activity by dopaminergic functional polymorphisms contributes to a predisposition to alcohol misuse in early adolescence. Psychological Medicine.

[B4-ejihpe-16-00152] Barnwell S. S., Earleywine M., Wilcox R. (2006). Cannabis, motivation, and life satisfaction in an internet sample. Substance Abuse Treatment, Prevention, and Policy.

[B5-ejihpe-16-00152] Bender R., Lange S. (2001). Adjusting for multiple testing—When and how?. Journal of Clinical Epidemiology.

[B6-ejihpe-16-00152] Beyer E., Poudel G., Antonopoulos S., Thomson H., Lorenzetti V. (2024). Brain reward function in people who use cannabis: A systematic review. Frontiers in Behavioral Neuroscience.

[B7-ejihpe-16-00152] Bloomfield M. A., Ashok A. H., Volkow N. D., Howes O. D. (2016). The effects of Δ9-tetrahydrocannabinol on the dopamine system. Nature.

[B8-ejihpe-16-00152] Bloomfield M. A., Morgan C. J., Kapur S., Curran H. V., Howes O. D. (2014). The link between dopamine function and apathy in cannabis users: An [18F]-DOPA PET imaging study. Psychopharmacology.

[B9-ejihpe-16-00152] Budney A. J. (2019). An update on cannabis use disorder with comment on the impact of policy related to therapeutic and recreational cannabis use. European Archives of Psychiatry and Clinical Neuroscience.

[B10-ejihpe-16-00152] Butterworth P., Slade T., Degenhardt L. (2014). Factors associated with the timing and onset of cannabis use and cannabis use disorder: Results from the 2007 Australian national survey of mental health and well-being. Drug and Alcohol Review.

[B11-ejihpe-16-00152] Büchel C., Peters J., Banaschewski T., Bokde A. L. W., Bromberg U., Conrod P. J., Flor H., Papadopoulos D., Garavan H., Gowland P., Heinz A., Walter H., Ittermann B., Mann K., Martinot J., Paillère-Martinot M., Nees F., Paus T., Pausova Z., Knutson B. (2017). Blunted ventral striatal responses to anticipated rewards foreshadow problematic drug use in novelty-seeking adolescents. Nature Communications.

[B12-ejihpe-16-00152] Caballero A., Tseng K. Y. (2012). Association of cannabis use during adolescence, prefrontal CB1 receptor signaling, and schizophrenia. Frontiers in Pharmacology.

[B13-ejihpe-16-00152] Cao Z., Bennett M., Orr C., Icke I., Banaschewski T., Barker G. J., Bokde A. L. W., Bromberg U., Büchel C., Quinlan E. B., Desrivières S., Flor H., Frouin V., Garavan H., Gowland P., Heinz A., Ittermann B., Martinot J., Nees F., IMAGEN Consortium (2019). Mapping adolescent reward anticipation, receipt, and prediction error during the monetary incentive delay task. Human Brain Mapping.

[B14-ejihpe-16-00152] Cassidy C. M., Lepage M., Harvey P.-O., Malla A. (2012). Cannabis use and anticipatory pleasure as reported by subjects with early psychosis and community controls. Schizophrenia Research.

[B15-ejihpe-16-00152] Collins A. C., Lekkas D., Struble C. A., Trudeau B. M., Jewett A. D., Griffin T. Z., Nemesure M. D., Price G. D., Heinz M. V., Nepal S., Pillai A., Mackin D. M., Campbell A. T., Budney A. J., Jacobson N. C. (2024). From mood to use: Using ecological momentary assessments to examine how anhedonia and depressed mood impact cannabis use in a depressed sample. Psychiatry Research.

[B16-ejihpe-16-00152] Connor J. P., Stjepanović D., Le Foll B., Hoch E., Budney A. J., Hall W. D. (2021). Cannabis use and cannabis use disorder. Nature Reviews Disease Primers.

[B17-ejihpe-16-00152] Cope L. M., Martz M. E., Hardee J. E., Zucker R. A., Heitzeg M. M. (2019). Reward activation in childhood predicts adolescent substance use initiation in a high-risk sample. Drug and Alcohol Dependence.

[B18-ejihpe-16-00152] Di Nicola M., Ferri V. R., Moccia L., Panaccione I., Strangio A. M., Tedeschi D., Grandinetti P., Callea A., De-Giorgio F., Martinotti G., Janiri L. (2017). Gender differences and psychopathological features associated with addictive behaviors in adolescents. Frontiers in Psychiatry.

[B19-ejihpe-16-00152] Dubol M., Trichard C., Leroy C., Sandu A., Rahim M., Granger B., Tzavara E. T., Karila L., Martinot J., Artiges E. (2018). Dopamine transporter and reward anticipation in a dimensional perspective: A multimodal brain imaging study. Neuropsychopharmacology.

[B20-ejihpe-16-00152] European School Survey Project on Alcohol and Other Drugs (ESPAD) (2024). ESPAD 2024: Key findings.

[B21-ejihpe-16-00152] European Union Drugs Agency (EUDA) (2026). European drug report 2026: Trends and developments.

[B22-ejihpe-16-00152] Freeman T. P., Craft S., Wilson J., Stylianou S., ElSohly M., Di Forti M., Lynskey M. T. (2021). Changes in delta-9-tetrahydrocannabinol (THC) and cannabidiol (CBD) concentrations in cannabis over time: Systematic review and meta-analysis. Addiction.

[B23-ejihpe-16-00152] Gard D. E., Gard M. G., Kring A. M., John O. P. (2006). Anticipatory and consummatory components of the experience of pleasure: A scale development study. Journal of Research in Personality.

[B24-ejihpe-16-00152] Gates P. J. (2016). Psychosocial interventions for cannabis use disorder. Cochrane Database of Systematic Reviews.

[B25-ejihpe-16-00152] Hambly Lapointe C., Nosko L., Crocker C. E., Tibbo P. G. (2026). Influence of cannabis potency on mental health outcomes among adolescents and young adults: Scoping review. BJPsych Open.

[B26-ejihpe-16-00152] Haney M., Vallée M., Fabre S., Collins Reed S., Zanese M., Campistron G., Arout C. A., Foltin R. W., Cooper Z. D., Kearney-Ramos T., Metna M. (2023). Signaling-specific inhibition of the CB1 receptor for cannabis use disorder: Phase 1 and phase 2a randomized trials. Nature Medicine.

[B27-ejihpe-16-00152] Heinze G., Wallisch C., Dunkler D. (2018). Variable selection—A review and recommendations for the practicing statistician. Biometrical Journal. Biometrische Zeitschrift.

[B28-ejihpe-16-00152] Hinckley J. D., Conner B. T., Mauch R., Arkfeld P. A., Bhatia D., Smith E. E., Svoboda E., Singh M. K. (2024). A systematic review: Investigating biomarkers of anhedonia and amotivation in depression and cannabis use. JAACAP Open.

[B29-ejihpe-16-00152] Hirvonen J., Goodwin R. S., Li C. T., Terry G. E., Zoghbi S. S., Morse C., Pike V. W., Volkow N. D., Huestis M. A., Innis R. B. (2012). Reversible and regionally selective downregulation of brain cannabinoid CB1 receptors in chronic daily cannabis smokers. Molecular Psychiatry.

[B30-ejihpe-16-00152] Hjorthøj C., Posselt C. M., Nordentoft M. (2021). Development over time of the population-attributable risk fraction for cannabis use disorder in schizophrenia in Denmark. JAMA Psychiatry.

[B31-ejihpe-16-00152] Hurd Y. L. (2025). An update on the behavioral and neurobiological effects of cannabis use in adolescents: A translational perspective. American Journal of Psychiatry.

[B32-ejihpe-16-00152] Kangas B. D., Deshpande H. U., Withey S. L., Spealman R. D., Bergman J., Kohut S. J. (2025). Chronic Δ9-tetrahydrocannabinol exposure in adolescent nonhuman primates: Persistent abnormalities in economic demand and brain functional connectivity. Neuropsychopharmacology.

[B33-ejihpe-16-00152] Leventhal A. M., Cho J., Stone M. D., Barrington-Trimis J. L., Chou C.-P., Sussman S. Y., Riggs N. R., Unger J. B., Audrain-McGovern J., Strong D. R. (2017). Associations between anhedonia and marijuana use escalation across mid-adolescence. Addiction.

[B34-ejihpe-16-00152] Lopez-Vergara H. I., Jackson K. M., Meshesha L. Z., Metrik J. (2019). Dysregulation as a correlate of cannabis use and problem use. Addictive Behaviors.

[B35-ejihpe-16-00152] Macedo I., Paiva T. O., Pasion R., Daedelow L., Heinz A., Magalhães A., Banaschewski T., Bokde A. L. W., Desrivières S., Flor H., Grigis A., Garavan H., Gowland P., Brühl R., Martinot J., Martinot M. P., Artiges E., Nees F., Orfanos D. P., Winterer J. M. (2024). Light cannabis use and the adolescent brain: An 8-years longitudinal assessment of mental health, cognition, and reward processing. Psychopharmacology.

[B36-ejihpe-16-00152] Martino I., Santangelo G., Moschella D., Marino L., Servidio R., Augimeri A., Costabile A., Capoderose G., Cerasa A. (2018). Assessment of Snaith–Hamilton Pleasure Scale (SHAPS): The dimension of anhedonia in Italian healthy sample. Neurological Sciences.

[B37-ejihpe-16-00152] Martz M. E., Cope L. M., Hardee J. E., Brislin S. J., Weigard A., Zucker R. A., Heitzeg M. M. (2021). Subtypes of inhibitory and reward activation associated with substance use variation in adolescence: A latent profile analysis of brain imaging data. Cognitive, Affective, & Behavioral Neuroscience.

[B38-ejihpe-16-00152] Martz M. E., Trucco E. M., Cope L. M., Hardee J. E., Jester J. M., Zucker R. A., Heitzeg M. M. (2016). Association of marijuana use with blunted nucleus accumbens response to reward anticipation. JAMA Psychiatry.

[B39-ejihpe-16-00152] Mishra S., Mishra S., Rajmohan A., Rath S. (2026). Cannabis use disorder as a disorder of motivational dysregulation: A systematic review of clinical, neurocognitive, and treatment evidence. Journal of Addictive Diseases.

[B40-ejihpe-16-00152] Mittmann G., Thomas M. F., Steiner-Hofbauer V. (2025). The Snaith-Hamilton Pleasure Scale (SHAPS) and Dimensional Anhedonia Rating Scale (DARS) diverge in measuring anhedonia in a community sample of young adults. Psychiatry Research.

[B41-ejihpe-16-00152] Moccia L., Mazza M., Di Nicola M., Janiri L. (2018). The experience of pleasure: A perspective between neuroscience and psychoanalysis. Frontiers in Human Neuroscience.

[B42-ejihpe-16-00152] Murray R. M., Englund A., Abi-Dargham A., Lewis D., Di Forti M., Davies C., Sherif M., McGuire P., D’SOuza D. (2017). Cannabis-associated psychosis: Neural substrate and clinical impact. Neuropharmacology.

[B43-ejihpe-16-00152] Paus T., Keshavan M., Giedd J. N. (2008). Why do many psychiatric disorders emerge during adolescence?. Nature Reviews Neuroscience.

[B44-ejihpe-16-00152] Rubino T., Parolaro D. (2016). The impact of exposure to cannabinoids in adolescence: Insights from animal models. Biological Psychiatry.

[B45-ejihpe-16-00152] Sagar K. A., Gruber S. A. (2025). The complex relationship between cannabis use and mental health: Considering the influence of cannabis use patterns and individual factors. CNS Drugs.

[B46-ejihpe-16-00152] Skumlien M., Langley C., Lawn W., Voon V., Curran H. V., Roiser J. P., Sahakian B. J. (2021). The acute and non-acute effects of cannabis on reward processing: A systematic review. Neuroscience & Biobehavioral Reviews.

[B47-ejihpe-16-00152] Snaith R. P., Hamilton M., Morley S., Humayan A., Hargreaves D., Trigwell P. (1995). A scale for the assessment of hedonic tone: The Snaith–Hamilton Pleasure Scale. The British Journal of Psychiatry.

[B48-ejihpe-16-00152] Solmi M., Radua J., Olivola M., Croce E., Soardo L., de Pablo G. S., Shin J. I., Kirkbride J. B., Jones P., Kim J. H., Kim J. Y., Carvalho A. F., Seeman M. V., Correll C. U., Fusar-Poli P. (2022). Age at onset of mental disorders worldwide: Large-scale meta-analysis of 192 epidemiological studies. Molecular Psychiatry.

[B49-ejihpe-16-00152] Stratta P., Pacifico R., Riccardi I., Daneluzzo E., Rossi A. (2011). Anticipatory and consummatory pleasure: Validation study of the Italian version of the Temporal Experience of Pleasure Scale. Journal of Psychopathology.

[B50-ejihpe-16-00152] Stull S. W., Bertz J. W., Epstein D. H., Bray B. C., Lanza S. T. (2022). Anhedonia and substance use disorders by type, severity, and with mental health disorders. Journal of Addiction Medicine.

[B51-ejihpe-16-00152] Trøstheim M., Eikemo M., Meir R., Hansen I., Paul E., Kroll S. L., Garland E. L., Leknes S. (2020). Assessment of anhedonia in adults with and without mental illness: A systematic review and meta-analysis. JAMA Network Open.

[B52-ejihpe-16-00152] Tseng K. Y., Molla H. M. (2025). Cannabinoid CB1 receptor-sensitive neurodevelopmental processes and trajectories. Molecular Psychiatry.

[B53-ejihpe-16-00152] United Nations Office on Drugs and Crime (UNODC) (2025). World drug report 2025.

[B54-ejihpe-16-00152] Verweij K. J. H., Huizink A. C., Agrawal A., Martin N. G., Lynskey M. T. (2013). Is the relationship between early-onset cannabis use and educational attainment causal or due to common liability?. Drug and Alcohol Dependence.

[B55-ejihpe-16-00152] Volkow N. D., Hampson A. J., Baler R. D. (2017). Don’t worry, be happy: Endocannabinoids and cannabis at the intersection of stress and reward. Annual Review of Pharmacology and Toxicology.

[B56-ejihpe-16-00152] Volkow N. D., Michaelides M., Baler R. (2019). The neuroscience of drug reward and addiction. Physiological Reviews.

[B57-ejihpe-16-00152] Wagner F. A., Anthony J. C. (2002). From first drug use to drug dependence: Developmental periods of risk for dependence upon marijuana, cocaine, and alcohol. Neuropsychopharmacology.

[B58-ejihpe-16-00152] Wijayendran S. B., O’Neill A., Bhattacharyya S. (2018). The effects of cannabis use on salience attribution: A systematic review. Acta Neuropsychiatrica.

