# Supplementary material for: Reduced Anticipatory Pleasure in Early-Onset Severe Cannabis Use Disorder: A Retrospective, Cross-Sectional Study
Source: Eur J Investig Health Psychol Educ. 2026 Sep 27;16(10):152. doi: 10.3390/ejihpe16100152 (PMC13703479; doi:10.3390/ejihpe16100152)

File S1. Participant flow.

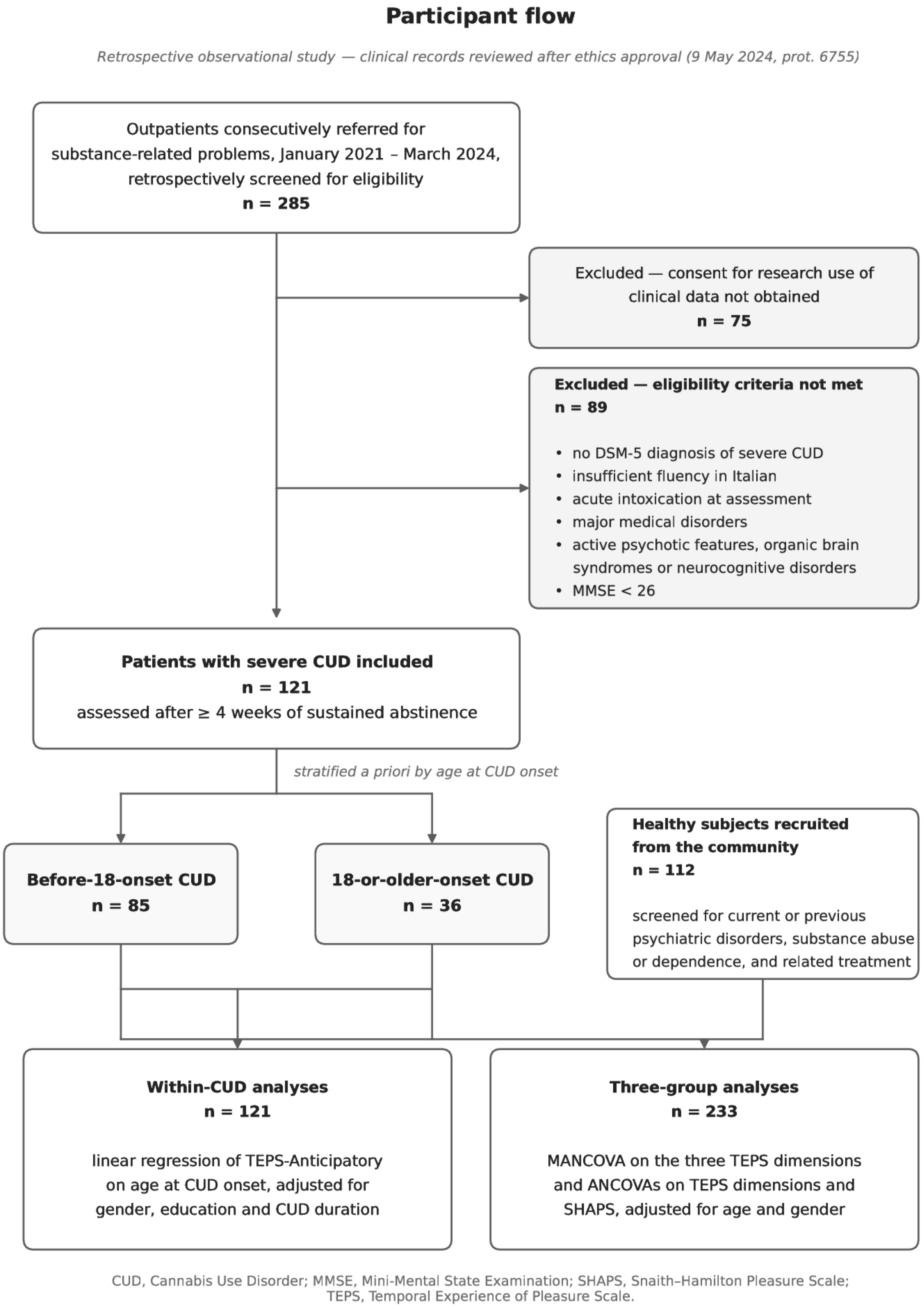

Supplement: Supplementary file 1 [file ejihpe-16-00152-s001.zip › File S1.pdf]
