# Supplementary material for: Reduced Anticipatory Pleasure in Early-Onset Severe Cannabis Use Disorder: A Retrospective, Cross-Sectional Study
Source: Eur J Investig Health Psychol Educ. 2026 Sep 27;16(10):152. doi: 10.3390/ejihpe16100152 (PMC13703479; doi:10.3390/ejihpe16100152)
Supplement: Supplementary file 1 [file ejihpe-16-00152-s001.zip › File S2.pdf]

File S2. Between-group differences in TEPS-Contextual Anticipatory and TEPS-Consummatory with age and gender as covariates (ANCOVA).

**ANCOVA Outcome**	**Effect**	**df**	**F**	***p***	**η^2p**
**TEPS-Contextual Anticipatory**	Group	2, 228	0.42	0.659	0.004
	Covariate				
	Age	1, 228	0.06	0.812	<0.001
	Gender	1, 228	2.01	0.158	0.009
**TEPS-Consummatory**	Group	2, 228	1.64	0.196	0.014
	Covariate				
	Age	1, 228	0.04	0.845	<0.001
	Gender	1, 228	0.03	0.853	<0.001

Abbreviations: ANCOVA, Analysis of Covariance; CUD, Cannabis Use Disorder; df, degrees of freedom; *p*, unadjusted statistical significance; η^2p, partial eta squared. N=233.
