# Supplementary material for: Reduced Anticipatory Pleasure in Early-Onset Severe Cannabis Use Disorder: A Retrospective, Cross-Sectional Study
Source: Eur J Investig Health Psychol Educ. 2026 Sep 27;16(10):152. doi: 10.3390/ejihpe16100152 (PMC13703479; doi:10.3390/ejihpe16100152)
Supplement: Supplementary file 1 [file ejihpe-16-00152-s001.zip › File S3.pdf]

File S3. Sensitivity analyses of between-group differences in hedonic measures additionally adjusted for educational level.

MANCOVA (TEPS)

**Effect**	**Wilks' Lambda**	**df**	**F**	***p***
Group	0.77	6, 450	10.63	**<0.001**
Covariate				
Age	0.99	3, 225	0.21	0.894
Gender	0.98	3, 225	1.40	0.244
Education	0.99	3, 225	0.95	0.419

ANCOVA

**Outcome**	**Effect**	**df**	**F**	***p***	**η^2p**
**TEPS-Anticipatory**	Group	2, 227	20.77	**<0.001**	0.155
	Covariate				
	Age	1, 227	0.02	0.881	<0.001
	Gender	1, 227	2.63	0.106	0.011
	Education	1, 227	1.41	0.238	0.006
**TEPS-Contextual Anticipatory**	Group	2, 227	0.47	0.628	0.004
	Covariate				
	Age	1, 227	0.09	0.760	<0.001
	Gender	1, 227	2.05	0.154	0.009
	Education	1, 227	0.17	0.682	0.001
**TEPS-Consummatory**	Group	2, 227	1.24	0.293	0.011
	Covariate				
	Age	1, 227	0.01	0.923	<0.001
	Gender	1, 227	0.03	0.872	<0.001
	Education	1, 227	0.29	0.586	0.001
**SHAPS**	Group	2, 227	46.18	**<0.001**	0.289
	Covariate				
	Age	1, 227	<0.001	0.979	<0.001
	Gender	1, 227	3.09	0.081	0.013
	Education	1, 227	0.01	0.915	<0.001

**Adjusted pairwise comparisons**	**MD (SE)**	$p^{\text{Bonferroni}}$
**TEPS-Anticipatory**		
Before-18 vs. 18-or-older-onset CUD	−5.09 (1.33)	**<0.001**
Before-18-onset CUD vs. HS	−7.17 (1.14)	**<0.001**
18-or-older-onset CUD vs. HS	−2.08 (1.35)	0.370
**SHAPS**		
Before-18 vs. 18-or-older-onset CUD	0.35 (0.39)	1.000
Before-18-onset CUD vs. HS	3.09 (0.34)	**<0.001**
18-or-older-onset CUD vs. HS	2.73 (0.40)	**<0.001**

Significant results are shown in **bold** characters. Bonferroni-adjusted *p*-values are reported for pairwise comparisons, which were performed and reported only for outcomes showing a significant group effect in the ANCOVAs. Abbreviations: ANCOVA, Analysis of Covariance; CUD, Cannabis Use Disorder; df, degrees of freedom; HS, Healthy Subjects; MANCOVA, Multivariate Analysis of Covariance; MD, Mean Difference; *p*, statistical significance; SE, Standard Error; SHAPS, Snaith-Hamilton Pleasure Scale; TEPS, Temporal Experience of Pleasure Scale; η^2p, partial eta squared. N=233.
