# Supplementary material for: Reduced Anticipatory Pleasure in Early-Onset Severe Cannabis Use Disorder: A Retrospective, Cross-Sectional Study
Source: Eur J Investig Health Psychol Educ. 2026 Sep 27;16(10):152. doi: 10.3390/ejihpe16100152 (PMC13703479; doi:10.3390/ejihpe16100152)
Supplement: Supplementary file 1 [file ejihpe-16-00152-s001.zip › File S4.pdf]

File S4. Exploratory correlations between overall anhedonia and hedonic–motivational dimensions.

Measure	**SHAPS**	
	r	***p***
**TEPS-Anticipatory**	−0.322	**<0.001**
**TEPS-Contextual Anticipatory**	−0.024	0.710
**TEPS-Consummatory**	−0.010	0.882
**TEPS-Total**	−0.212	**0.001**

Significant results in **bold** characters. Abbreviations: *p*, p-value; *r*, Pearson's correlation coefficient; SHAPS, Snaith-Hamilton Pleasure Scale; TEPS, Temporal Experience of Pleasure Scale. N=233.
